# Subregional Nowcasts of Seasonal Influenza Using Search Trends

**DOI:** 10.2196/jmir.7486

**Published:** 2017-11-06

**Authors:** Sasikiran Kandula, Daniel Hsu, Jeffrey Shaman

**Affiliations:** ^1^ Department of Environmental Health Sciences Columbia University New York, NY United States; ^2^ Department of Computer Science Columbia University New York, NY United States

**Keywords:** human influenza, classification and regression trees, nowcasts, infodemiology, infoveillance, surveillance

## Abstract

**Background:**

Limiting the adverse effects of seasonal influenza outbreaks at state or city level requires close monitoring of localized outbreaks and reliable forecasts of their progression. Whereas forecasting models for influenza or influenza-like illness (ILI) are becoming increasingly available, their applicability to localized outbreaks is limited by the nonavailability of real-time observations of the current outbreak state at local scales. Surveillance data collected by various health departments are widely accepted as the reference standard for estimating the state of outbreaks, and in the absence of surveillance data, nowcast proxies built using Web-based activities such as search engine queries, tweets, and access of health-related webpages can be useful. Nowcast estimates of state and municipal ILI were previously published by Google Flu Trends (GFT); however, validations of these estimates were seldom reported.

**Objective:**

The aim of this study was to develop and validate models to nowcast ILI at subregional geographic scales.

**Methods:**

We built nowcast models based on autoregressive (autoregressive integrated moving average; ARIMA) and supervised regression methods (Random forests) at the US state level using regional weighted ILI and Web-based search activity derived from Google's Extended Trends application programming interface. We validated the performance of these methods using actual surveillance data for the 50 states across six seasons. We also built state-level nowcast models using state-level estimates of ILI and compared the accuracy of these estimates with the estimates of the regional models extrapolated to the state level and with the nowcast estimates published by GFT.

**Results:**

Models built using regional ILI extrapolated to state level had a median correlation of 0.84 (interquartile range: 0.74-0.91) and a median root mean square error (RMSE) of 1.01 (IQR: 0.74-1.50), with noticeable variability across seasons and by state population size. Model forms that hypothesize the availability of timely state-level surveillance data show significantly lower errors of 0.83 (0.55-0.23). Compared with GFT, the latter model forms have lower errors but also lower correlation.

**Conclusions:**

These results suggest that the proposed methods may be an alternative to the discontinued GFT and that further improvements in the quality of subregional nowcasts may require increased access to more finely resolved surveillance data.

## Introduction

Seasonal influenza infections are estimated to occur in 5% to 10% of the adult population worldwide annually, with higher attack rates in children and older adults [1,2]. In the United States, influenza accounts for about 1.2 deaths per 100,000 people, with considerable interseasonal variability [3]. Municipal and state health departments rely on surveillance data throughout the influenza season, typically October through May in the United States, to track the progress of the season and to coordinate vaccination and treatment activities among hospitals, health care providers, and public health agencies. To support these efforts, the Centers for Disease Control and Prevention (CDC) disseminates weekly virologic and outpatient incidence data for influenza-like illness (ILI) at national and regional levels [4,5].

Several methods have been proposed to complement CDC's ILI, with estimates based on search queries [6-11], tweets [12,13], Wikipedia access logs [14,15], other public-generated content [16-18], and a combination of these proxies [19]. In addition to providing more timely estimates of outbreak progression, these data sources can be used to develop ILI estimates at the more localized subregional geographic resolutions at which public ILI data are limited or unavailable. As more effective and targeted interventions can be designed through a more local view of the outbreak, these subregional estimates, if accurate and reliable, are more actionable.

Google Flu Trends (GFT) [6] generated one of the more widely available estimates of ILI at regional and subregional levels using trends in Web-based search queries; however, production of GFT estimates was discontinued in August 2015 [20]. Instead, through Google's Extended Trends (GET) application programming interface (API), researchers are now permitted access to underlying Google search trends data and may build their own models to estimate ILI. The original GFT approach models CDC ILI as a linear function of search query frequency aggregated as a single variable. More recent work [7,21] demonstrated improved accuracy when individual query terms were retained as independent variables in the linear model, and further gain was reported with alternate models that allow for nonlinear and temporal relationships between the queries. A related study modeled ILI at week *w* on both autoregressive lags of n-weeks and search volume of 100 selected terms during week *w* [8,22].

Whereas these studies are encouraging, these models were developed and validated at US national level where the response variable, ILI, is available. Extrapolation of these national models to subregional resolutions where CDC ILI is not publicly available may yield nowcasts of limited accuracy. The GFT team is yet to publish the methodology used to generate nowcasts at subregional scales, and there have been few validation studies for GFT estimates at subregional levels [23,24].

In this paper, we propose methods to nowcast ILI at the subregional level using GET. These methods were applied retrospectively to generate nowcasts in 50 US states for six seasons, report the accuracy of different model forms, and compare these with GFT as published. It was observed that accurate nowcasts of subregional ILI may not be possible using models developed at the regional level; rather, subregional ILI nowcast models must be developed using subregional ILI data.

## Methods

### Overview

To build nowcast models at the US state level, random forest regression models were first built at the regional level (as defined by the US Department of Health and Human Services, HHS [25]). In these initial models, HHS regional weighted ILI, as reported by the CDC, was the response variable, and queries with search patterns correlated with ILI were explanatory variables. A 1-week ahead forecast of an autoregressive model fit on regional ILI was included as an additional explanatory variable. These regional-level models were then applied, or extrapolated, at the subregional scale. Specifically, the fit models were used with state-level explanatory variables to estimate ILI at the state level.

Independently, state-level nowcast models were built using CDC-provided state-level estimates of ILI as the response variable. These state-level ILI estimates are not publicly available and were provided for this study on request. The error of the state-level nowcast estimates made using these state models was then compared with the estimates of the regional models extrapolated to the state level.

### Google Extended Trends (GET) Application Programming Interface

The GET API allows users to retrieve timeline data of the probability that a specified term is queried during a search session. Additional parameters allow specification of geographical (country, state, etc) and temporal (daily, weekly, etc) granularity and period of interest. Query probability is calculated on a random sample of 10% to 15% of all searches; terms whose search volume does not meet a minimum threshold are considered private, and their probabilities are reported as 0. Data updates are made daily and historical trends from January 2004 are available. Hence, nowcast models developed using GET can provide ILI estimates for at least one additional week over CDC ILI data, which are released with a 5- to 11-day lag.

In this study, as we were interested in state-level nowcasts, *state* was used as the geographical resolution and a *weekly* periodicity to be consistent with CDC ILI and GFT, both of which are weekly ILI estimates. We refer to logit transformed time series of term *t* as the query fraction of *t*, that is, *qf(t, s, w) = log (z/(1-z))* where *z* is the probability that a query from state *s* during week *w* is for term *t*. GET does not provide separate query fractions at the HHS regional level. Therefore, the query fraction for a term from an HHS region was calculated as a population weighted mean of the query fractions for the term from states within the region. This choice of transformation was informed by previous work, which found that with logit transformation, the relation between raw query fractions and ILI becomes approximately linear and model performance improves [7].

### Feature Identification

Queries highly correlated with CDC ILI were identified using Google Correlate [26,27] for use as explanatory variables. Google Correlate returns 100 queries whose search trends are historically most highly correlated (Pearson correlation coefficient) with a given target time series data. ILI at US national and 10 HHS regional levels from 2003-04 through 2014-15 influenza season was used as target time series. Significant overlap was observed in the queries identified using the different target time series. Query terms identified by Zhang [28] and influenza-related entities extracted from Freebase [29], were appended to the list of correlates.

While examining the query fractions for terms related to ILI, it was found that some terms, which have considerable search activity at the national level, often have little-to-no activity at the state level and are reported as 0 (Multimedia Appendix 1; Figure S1), possibly because of the sampling and threshold criteria used in GET. Hence, the explanatory variables at the state level are sparse. To improve the quality of the data, a form of inheritance where a state inherits the query fraction of a term at the regional level when the state-level query fraction is zero was employed: *qf(t, s, w) = qf(t, r, w)*, where *s* ∊ *r*, and *r* designates HHS region. That is, in the absence of additional information, we assume users in all states of a region search for a term with the same probability. As this would not eliminate all zeros, the remaining zeros were replaced with a very small value, 1e-12, before applying the logit transformation. Sensitivity analysis showed that the results were not sensitive to the choice of the replacement (Multimedia Appendix 1; Figure S2).

### Autoregressive Integrated Moving Average

Lampos et al [7] have found that simple autoregressive integrated moving average (ARIMA) models [30-32] using search trends data can generate reasonable nowcast estimates for ILI at the US national level. Similarly, Broniatowski et al [33,34] have demonstrated the utility of ARIMA models that use tweets and query data at a few subregional locations. ARIMA models are specified with three parameters, the order of the autoregressive component (a), the degree of differencing (d), and the order of the moving average component (q).

**Figure 1:**
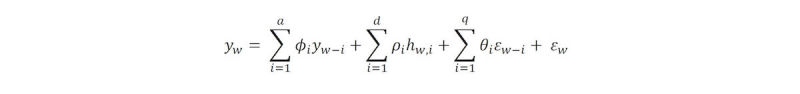
Autoregressive integrated moving average (ARIMA) formulation.

In Figure 1, *ф*, *θ*, and *ρ* are to be learned during model fitting. A method described by Hyndman and Khandakar [35,36] was used to search parameter space and to identify a set of parameters that provides good model fit, and the ARIMA models built at different times (*w*) and for different regions were allowed to use different parameters.

### Random Forest

Random forest is a decision tree–based ensemble supervised learner that can be used for regression [37-39]. Specifically, given a dataset of *n* instances *D=(X, Y)=(x*_ip,_*y*_i_*)*, where *Y* is a continuous response variable, and the feature set *X=(X*_1_*, X*_2,_*...,X*_p_*)* of *p* explanatory variables (ie, *x*_ip_ is the value of feature *j* for instance *i*), a supervised learning algorithm uses *D* to learn a function *ḟ* such that *Ẏ= ḟ(X)* and Ẏ minimizes some loss function with respect to *Y*. The function *ḟ* can then be used to estimate *ẏ*_0_ for an instance *x*_0_=(*x*_01_*, x*_02_*, …,x*_op_) whose response is unknown.

Decision tree–based methods split the feature space along an explanatory variable and learn separate fits, *ḟ* for each subspace. Ensemble methods build multiple decision trees, each tree on a dataset D^*^, a random sampling with replacement of *n* instances from *D*. Random forests are ensemble decision trees that also exclude a random subset of explanatory variables while learning. Random forests are suitable for nonlinear problems with large feature sets and have been found to offer superior accuracy in multiple domains.

In this study, the randomForest [45] package in R [46] (R Project for Statistical Computing) was used to build the models.

### Model Formulation

The model is described in detail in Multimedia Appendix 1. To summarize, let *y*_1:w_^r^ denote the logit transformed ILI observations for region *r* through week *w;* and *X*_1:v_^r^ a query fraction matrix of logit transformed query fractions at HHS region *r* for all terms in the feature set (columns) during weeks 1 through week *v* (rows). Note that *v>w*. We fit an ARIMA model on *y*_1:w_^r^ forecast ahead for weeks *w+1* to *v* and add the ARIMA result as an explanatory variable to *X*_1:v_^r^. With this modified matrix as the predictor and (*y*_1:w_^r^ )^T^ as the response, we train a random forest model for region *r* at week *w*, *ḟ*_w_^r^. To nowcast ILI for a state *s* in region *r*, we append region *r*'s ARIMA results to the state's query fraction matrix *X*_1:v_^s^, and use this as a test set with *ḟ*_w_^r^.

### Validation

State level ILI counts (per 100,000 patient visits) from 2000-01 to 2010-11 season were provided by CDC following a data request. These counts were considered as the true values to validate the estimates from the model described above. As GET data were only available from January 2004, the last six of the seven overlapping flu seasons (*Morbidity and Mortality Weekly Report* [40], MMWR, week 40 through MMWR week 39 of the next calendar year), that is, 2005-06 to 2010-11 were used for validation. To generate nowcasts for any given week, only data that would have been available if nowcasts were being generated in real time were used, thus allowing for an out-of-sample validation of the estimates.

**Figure 2:**
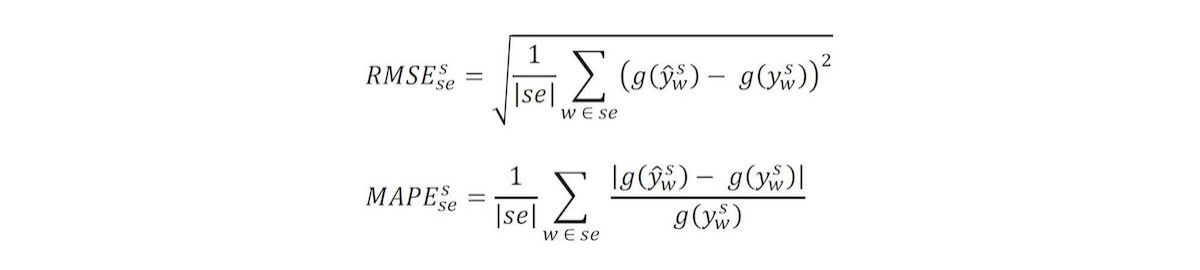
Formulation for two error measures: root mean square error (RMSE) and mean absolute proportion error (MAPE).

For each state during each of the six seasons, the Pearson correlation coefficient (COR), root mean square error (RMSE), and mean absolute proportion error (MAPE) were calculated. In Figure 2, *y*_w_^s^ is the true ILI for state *s* at week *w*, *ẏ*_w_^s^ the corresponding nowcast, *w* ∊ *se* the weeks in a given flu season, and *g()* is the inverse logit transformation. Although nowcast estimates up to 2 weeks ahead are sometimes possible using ARIMA and GET, only 1-week ahead estimates were used in this error analysis.

### Alternate Model Forms

To generate nowcasts for a state, the model trained with its corresponding regional data was extrapolated to the state level. For this extrapolation, the model formulation, described above and trained using regional ILI as the response variable, was applied using an ARIMA fit of regional ILI and state GET query fractions as explanatory variables. We refer to this form as RRS. Two other alternate forms were explored: RR0, where the state's ILI estimate is simply its region's ARIMA estimate, and RRR, where the state's GET query fractions were replaced with the query fractions for its parent region.

The accuracy of RRS relative to RR0 was indicative of value added by GET and random forests, and of RRS relative to RRR as value added through the use of more localized GET data. As GFT was published during the six seasons being used for validation, the performance of these three model forms were also compared against GFT.

### Alternate Model Forms: State ILI as Response

The three model forms described above were built with regional ILI as the response variable. As regional ILI is released weekly by the CDC, these models are suitable for real-time operational nowcasts. Although estimates of subregional ILI are not publicly available, state and municipal health agencies do have these estimates for internal use, and it is worthwhile to develop and test model forms that would be possible with subregional ILI.

Four additional model forms were defined: SS0, a simple ARIMA model fit on state ILI; SRR and SRS, which are similar to RRR and RRS, respectively, except for the response variable used for training; and SSS, which does not directly use any regional information. Please see Multimedia Appendix 1 for more formal specification of these four types.

To compare the different model forms and to check that the differences were statistically significant, we used a Friedman rank-sum test [41,42] followed by a Nemenyi test [43,44]. The Friedman test is a nonparametric test that does not assume normality. It ranks the different model forms on each test attempt, a state-season combination and uses the rank to test whether model forms are different. The Nemenyi test, a post hoc test for Friedman, checks for statistically significant differences between each pair of model forms.

## Results

**Figure 3:**
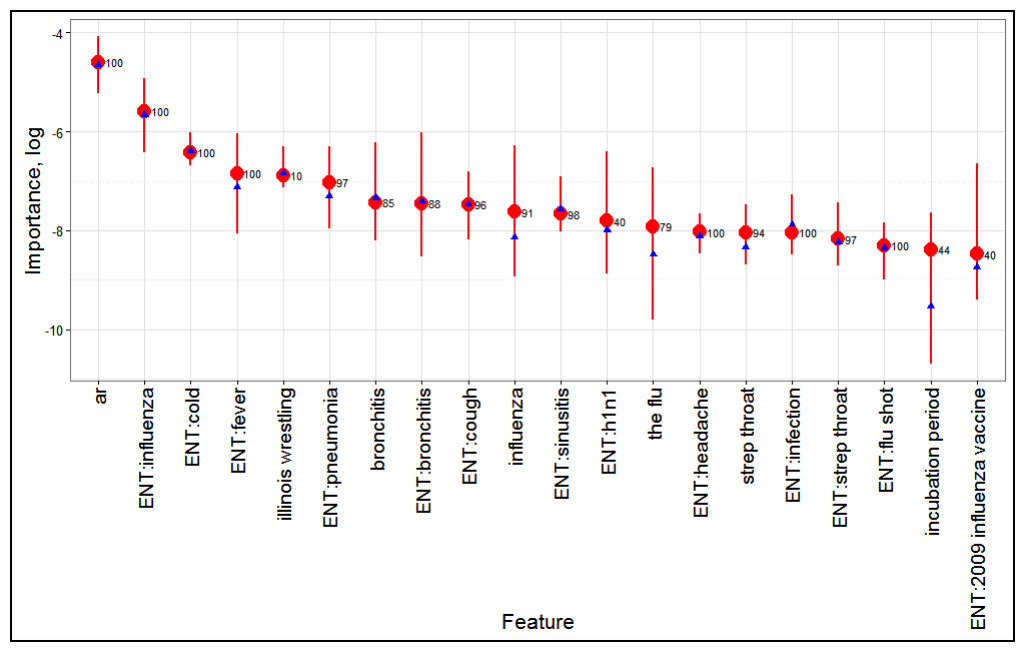
Top 20 features by importance as determined by random forest models built at regional level. The dot and whiskers in red show the median and interquartile range (IQR), respectively, whereas the blue point is the mean. The label shows the percentage of models in which the feature was used (n=3130). ar refers to the autoregressive integrated moving average (ARIMA) component. Features prefixed by ENT are entities identified using Freebase.

Of the explanatory variables used in the RRS models, the ARIMA component (*ar*) ranks highest followed by a good number of entities from Freebase (see Figure 3). Across all seasons and states, the RRS models were found to have a reasonably high median correlation of 0.84 (interquartile range [IQR]: 0.74-0.91; Table 1). When stratified by population size, states with larger population sizes had significantly higher median correlations than those with small population sizes. Significant variability across seasons was also observed. States with large populations sizes were also found to have lower median errors (RMSE and MAPE), but there does not seem to be much difference between low- and medium-sized states.

Although the correlation of the RRS models was encouraging, GFT estimates have better median measures overall and across almost all disaggregated groups. Google has not published their method to estimate ILI at subregional levels, and it is not clear whether GFT estimates benefited from a fuller access to trends data or whether the performance gain was solely methodological.

**Table 1:** Median (interquartile range), Pearson correlation coefficient (COR), root mean square error (RMSE), and mean absolute proportion error (MAPE) for RRS, RR0, RRR models, and Google Flu Trends (GFT). Results are stratified by state population size and season.

Measure			RRS, median (interquartile range)	RR0, median (interquartile range)	RRR, median (interquartile range)	GFT^a^, median (interquartile range)
**COR**^b^						
	Overall		0.85 (0.74-0.91)	0.83 (0.7-0.9)	0.86 (0.75-0.91)	0.89 (0.8-0.94)
	**Population size (millions)**					
		0-2 (n=14)	0.79 (0.64-0.87)	0.76 (0.62-0.86)	0.81 (0.67-0.88)	0.83 (0.72-0.91)
		2-5 (n=14)	0.84 (0.72-0.89)	0.82 (0.7-0.89)	0.84 (0.75-0.90)	0.9 (0.81-0.94)
		5-7.5 (n=10)	0.84 (0.74-0.91)	0.82 (0.7-0.9)	0.86 (0.73-0.92)	0.89 (0.8-0.95)
		≥7.5 (n=12)	0.91 (0.85-0.93)	0.9 (0.84-0.93)	0.91 (0.86-0.94)	0.93 (0.86-0.96)
	**Season**					
		05-06	0.8 (0.62-0.85)	0.8 (0.62-0.85)	0.81 (0.64-0.87)	0.83 (0.71-0.88)
		06-07	0.82 (0.65-0.88)	0.8 (0.6-0.88)	0.82 (0.71-0.89)	0.83 (0.76-0.9)
		07-08	0.88 (0.81-0.92)	0.87 (0.79-0.92)	0.89 (0.82-0.93)	0.93 (0.87-0.96)
		08-09	0.75 (0.69-0.83)	0.71 (0.58-0.82)	0.78 (0.67-0.83)	0.81 (0.71-0.89)
		09-10	0.9 (0.85-0.93)	0.89 (0.8-0.93)	0.9 (0.85-0.93)	0.97 (0.94-0.98)
		10-11	0.89 (0.82-0.92)	0.88 (0.75-0.91)	0.89 (0.85-0.92)	0.89 (0.86-0.93)
**RMSE**^c^						
	Overall		0.99 (0.7-1.51)	1.06 (0.73-1.56)	0.97 (0.72-1.54)	0.93 (0.66-1.33)
	**Population size (millions)**					
		0-2 (n=14)	1.06 (0.69-1.58)	1.19 (0.73-1.62)	1.05 (0.72-1.6)	0.88 (0.63-1.29)
		2-5 (n=14)	1.21 (0.84-1.87)	1.33 (0.92-1.81)	1.22 (0.83-1.84)	1.02 (0.78-1.52)
		5-7.5 (n=10)	0.93 (0.65-1.21)	0.98 (0.72-1.33)	0.93 (0.61-1.14)	0.88 (0.67-1.48)
		≥7.5 (n=12)	0.87 (0.66-1.01)	0.85 (0.70-1.08)	0.88 (0.69-1.01)	0.87 (0.63-1.16)
	**Season**					
		05-06	0.93 (0.64-1.5)	0.92 (0.70-1.64)	0.93 (0.64-1.52)	0.88 (0.60-1.45)
		06-07	0.84 (0.56-1.16)	0.89 (0.57-1.16)	0.85 (0.5-1.1)	0.82 (0.52-1.13)
		07-08	1.08 (0.81-1.7)	1.06 (0.83-1.59)	0.99 (0.82-1.67)	1.09 (0.70-1.55)
		08-09	1.02 (0.77-1.47)	1.10 (0.79-1.48)	1.03 (0.79-1.55)	1.02 (0.79-1.41)
		09-10	1.31 (0.98-1.77)	1.40 (1.08-1.72)	1.28 (0.98-1.72)	1.05 (0.80-1.32)
		10-11	0.77 (0.59-1.16)	0.83 (0.61-1.26)	0.83 (0.59-1.15)	0.73 (0.64-1.20)
**MAPE**^d^ **(/1000)**						
	Overall		0.8 (0.43-1.75)	0.67 (0.42-1.54)	0.77 (0.43-1.62)	0.71 (0.44-1.51)
	**Population size (millions)**					
		0-2 (n=14)	0.9 (0.54-1.7)	0.77 (0.51-1.41)	0.84 (0.55-1.55)	0.76 (0.51-1.56)
		2-5 (n=14)	0.95 (0.48-1.79)	0.82 (0.44-1.65)	0.87 (0.45-1.71)	0.77 (0.41-1.48)
		5-7.5 (n=10)	0.65 (0.36-1.62)	0.59 (0.37-1.69)	0.63 (0.35-1.57)	0.68 (0.4-1.41)
		≥7.5 (n=12)	0.65 (0.34-1.64)	0.54 (0.3-1.34)	0.65 (0.33-1.5)	0.7 (0.43-1.54)
	**Season**					
		05-06	1.2 (0.46-3.06)	0.78 (0.47-2.77)	0.99 (0.49-2.72)	1.07 (0.56-2.67)
		06-07	0.97 (0.53-1.84)	0.92 (0.49-1.81)	0.91 (0.51-1.67)	0.88 (0.46-1.48)
		07-08	0.85 (0.5-1.67)	0.83 (0.49-1.64)	0.81 (0.51-1.51)	0.76 (0.5-1.57)
		08-09	0.82 (0.47-1.59)	0.67 (0.43-1.36)	0.84 (0.43-1.52)	0.71 (0.44-1.48)
		09-10	0.73 (0.36-1.96)	0.64 (0.4-1.83)	0.74 (0.36-1.96)	0.63 (0.43-1.17)
		10-11	0.49 (0.3-1.04)	0.48 (0.28-0.96)	0.48 (0.31-1.04)	0.61 (0.32-0.93)

^a^GFT: Google Flu Trends.

^b^COR: Pearson correlation coefficient.

^c^RMSE: root mean square error.

^d^MAPE: mean absolute percentage error.

**Table 2:** Mean rank and statistical significance from post hoc Nemenyi test. For each season-state combination, the model forms are ranked from best (rank=1) to worst (rank=4).

Model	COR^a^				RMSE^b^				MAPE^c^			
	Mean rank	GFT^d^	RRO	RRR	Mean rank	GFT	RRO	RRR	Mean rank	GFT	RRO	RRR
GFT	1.91				2.33				2.45			
RR0	3.07	<.001			2.75	<.001			2.24	.17		
RRR	2.38	<.001	<.001		2.41	.89	.01		2.43	.99	.25	
RRS	2.63	<.001	<.001	.1	2.51	.35	.09	.79	2.87	<.001	<.001	<.001

^a^COR: Pearson correlation coefficient.

^b^RMSE: root mean square error.

^c^MAPE: mean absolute percentage error.

^d^GFT: Google Flu Trends.

Table 2 shows the mean rank for the model forms along with the results of Friedman-Nemenyi tests for significance. Of the four estimates, the best performing (highest correlation or lowest error) is assigned a rank of 1, the worst a rank of 4, and an average across the different season-state combinations (n=300) is calculated. The results indicate the following: (1) For correlation, GFT has the best mean rank, followed by RRR, RRS, and RR0. However, the difference between RRR and RRS is not statistically significant; (2) the relative ordering of the mean ranks remains the same with RMSE, but the difference between RR0, RRR, and RRS is not statistically significant; and, (3) RR0 has the best rank with MAPE followed by GFT. The mean ranks of RRR and RRS are significantly higher.

Overall, the performance of the RRR models was comparable to the RRS models, which indicates that state-localized GET data, as used in the models described here, do not improve nowcast accuracy. Because RR0 lowers (degrades) correlation, does not alter RMSE and considerably lowers (improves) MAPE, the effect of ignoring GET data altogether remains uncertain.

**Figure 4:**
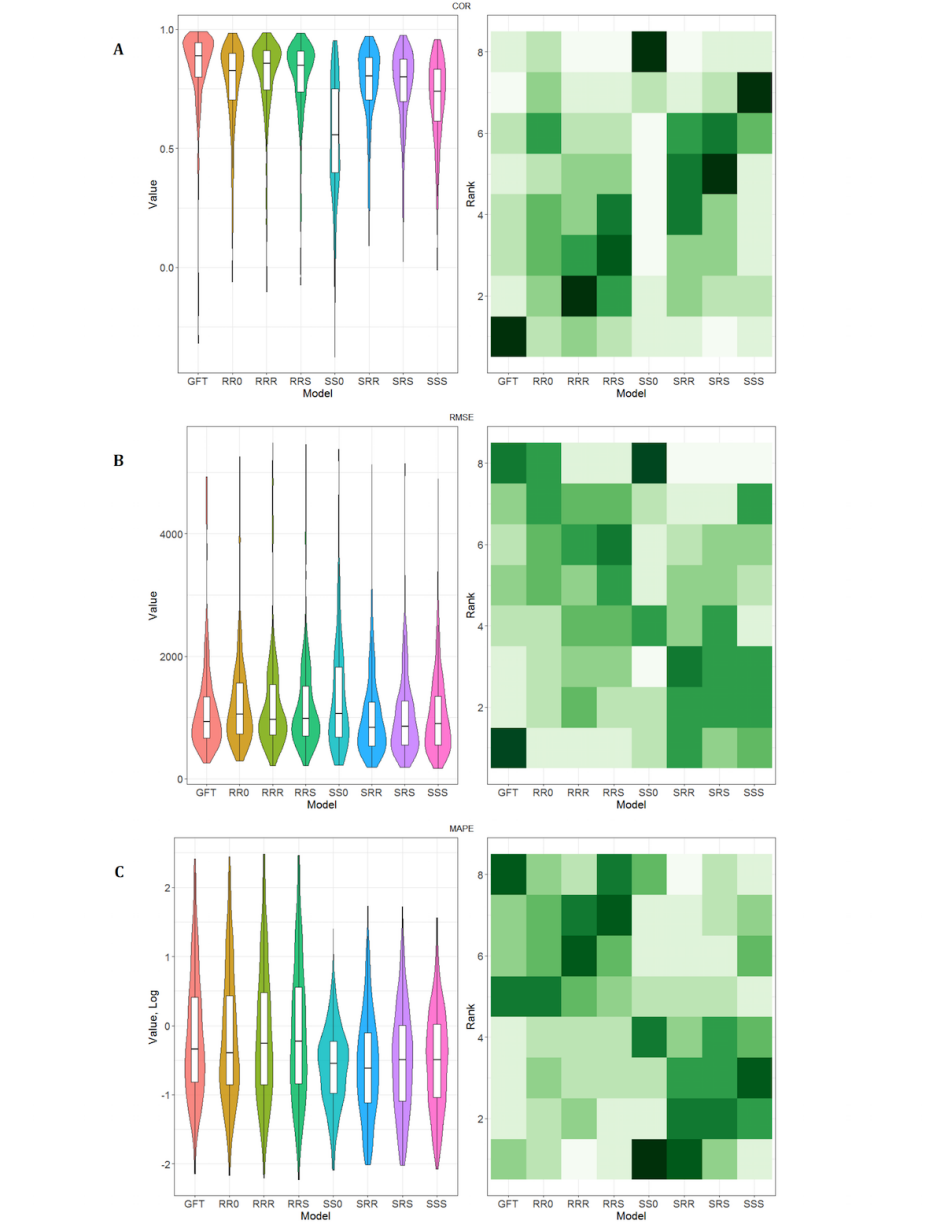
Measures observed with the different model forms A: Pearson correlation coefficient (COR); B: Root mean square error (RMSE); and C: Mean absolute percentage error (MAPE). Left: The box and whiskers show the median, interquartile range (IQR), and extrema (1.5×IQR) for each model form. The colored regions are violin plots showing probability density. Right: Heat map of the distribution of relative ranks of the models; more frequent ranks are darker.

**Figure 5:**
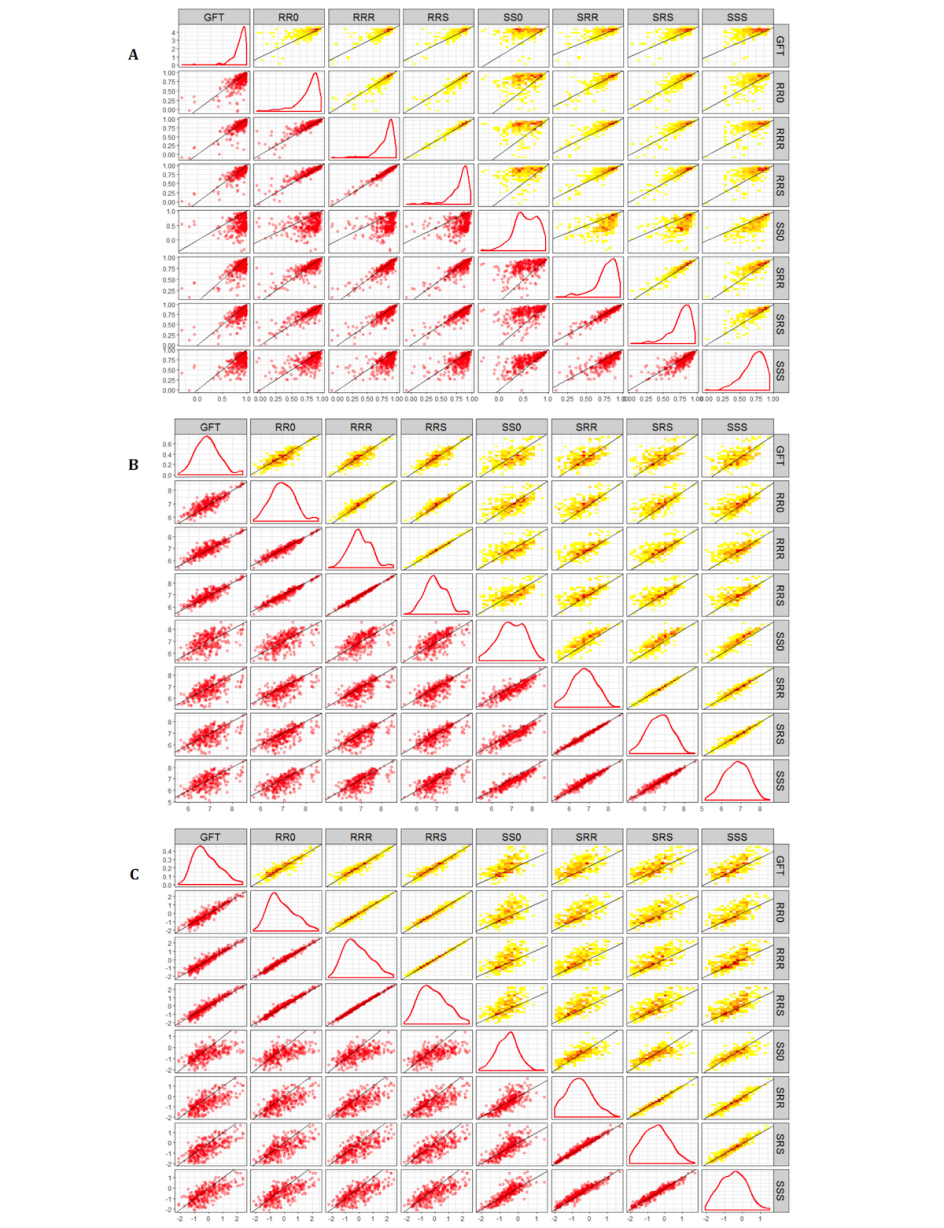
Pairwise plots for the model forms on the three measures forms A: Pearson correlation coefficient (COR); B: Root mean square error (RMSE); and C: Mean absolute percentage error (MAPE). The subpanels along the diagonal show density of the measure for the model form. Subpanels in the lower triangle are scatter plots (n=300) denoting a state-season. Points on or close to the black line (y=x) are state-seasons where the pair of model forms have similar measures (correlation or error). Subpanels in the upper triangle are heat maps of the counts of points in each two-dimensional (2D) grid of the plot area (low counts in yellow, high in red). For example, to compare the correlations of RRS and SS0, see the scatter plot in (5,4) or heat map in (4,5) of A.

Extending the comparison to model forms that are built using state ILI as the response variable (Table 3; Figures 4 and 5), a noticeable reduction was observed in errors. The median RMSE and MAPE (Figure 4) of the SRS, SRR, and SSS models are lower than GFT overall, in states with larger population, and in a majority of the seasons. There is also a clear improvement over their RR* counterparts (Figure 5). However, the median correlation of all four models is noticeably lower, especially for the SS0 models.

**Table 3:** Median (interquartile range), Pearson correlation coefficient (COR), root mean square error (RMSE), and mean absolute percentage error (MAPE) for Google Flu Trends (GFT), SS0, SRR, SRS, and SSS models. Results are stratified by state population and season.

Measure			GFT^a^, median (interquartile range)	SS0, median (interquartile range)	SRR, median (interquartile range)	SRS, median (interquartile range)	SSS, median (interquartile range)
**COR**^b^							
	Overall		0.89 (0.8-0.94)	0.56 (0.4-0.75)	0.8 (0.7-0.88)	0.8 (0.7-0.88)	0.74 (0.61-0.83)
	**Population size (millions)**						
		0-2 (n=14)	0.83 (0.72-0.91)	0.46 (0.31-0.66)	0.74 (0.57-0.82)	0.71 (0.56-0. 8)	0.62 (0.55-0.74)
		2-5 (n=14)	0.9 (0.81-0.94)	0.58 (0.42-0.76)	0.78 (0.72-0.87)	0.8 (0.72-0.85)	0.73 (0.66-0.81)
		5-7.5 (n=10)	0.89 (0.8-0.95)	0.51 (0.36-0.64)	0.83 (0.7-0.88)	0.81 (0.73-0.88)	0.75 (0.63-0.82)
		≥7.5 (n=12)	0.93 (0.86-0.96)	0.73 (0.48-0.85)	0.88 (0.79-0.92)	0.89 (0.8-0.92)	0.86 (0.72-0.91)
	**Season**						
		05-06	0.83 (0.71-0.88)	0.72 (0.56-0.85)	0.78 (0.68-0.86)	0.76 (0.62-0.86)	0.74 (0.66-0.86)
		06-07	0.83 (0.76-0.9)	0.75 (0.61-0.84)	0.8 (0.7-0.88)	0.8 (0.64-0.87)	0.8 (0.72-0.89)
		07-08	0.93 (0.87-0.96)	0.61 (0.47-0.77)	0.87 (0.78-0.92)	0.86 (0.78-0.9)	0.81 (0.73-0.86)
		08-09	0.81 (0.71-0.89)	0.37 (0.28-0.44)	0.7 (0.59-0.8)	0.74 (0.58-0.79)	0.57 (0.45-0.68)
		09-10	0.97 (0.94-0.98)	0.51 (0.39-0.73)	0.82 (0.75-0.89)	0.82 (0.74-0.89)	0.74 (0.63-0.85)
		10-11	0.89 (0.86-0.93)	0.47 (0.33-0.6)	0.82 (0.75-0.88)	0.81 (0.75-0.88)	0.71 (0.63-0.78)
**RMSE**^c^**(1e-3)**							
	Overall		0.93 (0.66-1.33)	1.07 (0.68-1.84)	0.84 (0.54-1.25)	0.86 (0.55-1.27)	0.9 (0.55-1.35)
	**Population size (millions)**						
		0-2 (n=14)	0.88 (0.63-1.29)	1.17 (0.61-1.92)	0.96 (0.55-1.47)	0.96 (0.62-1.49)	0.92 (0.58-1.44)
		2-5 (n=14)	1.02 (0.78-1.52)	1.37 (0.83-2.13)	1.04 (0.7-1.54)	1.11 (0.62-1.57)	1.11 (0.66-1.68)
		5-7.5 (n=10)	0.88 (0.67-1.48)	0.99 (0.66-1.79)	0.74 (0.49-1.07)	0.71 (0.51-1.14)	0.79 (0.55-1.24)
		≥7.5 (n=12)	0.87 (0.63-1.16)	0.91 (0.64-1.49)	0.69 (0.43-1.05)	0.67 (0.41-0.99)	0.74 (0.46-1.01)
	**Season**						
		05-06	0.88 (0.60-1.45)	0.81 (0.49-1.47)	0.71 (0.5-1.11)	0.68 (0.49-1.13)	0.64 (0.46-1.06)
		06-07	0.82 (0.52-1.13)	0.70 (0.48-1.02)	0.59 (0.43-0.88)	0.58 (0.42-0.94)	0.56 (0.41-0.83)
		07-08	1.09 (0.70-1.55)	1.36 (0.78-1.85)	0.91 (0.54-1.27)	0.95 (0.58-1.37)	0.97 (0.6-1.42)
		08-09	1.02 (0.79-1.41)	1.21 (0.92-1.98)	0.95 (0.69-1.31)	0.93 (0.67-1.26)	1.05 (0.78-1.4)
		09-10	1.05 (0.80-1.32)	1.91 (1.28-2.44)	1.34 (0.9-1.9)	1.37 (0.92-1.92)	1.53 (1.01-1.9)
		10-11	0.73 (0.64-1.20)	1.00 (0.73-1.62)	0.73 (0.5-1.04)	0.7 (0.51-1.1)	0.86 (0.58-1.16)
**MAPE**^d^							
	Overall		0.71 (0.44-1.51)	0.58 (0.38-0.8)	0.54 (0.33-0.9)	0.61 (0.34-1)	0.61 (0.35-1.02)
	**Population size (millions)**						
		0-2 (n=14)	0.76 (0.51-1.56)	0.68 (0.48-0.86)	0.76 (0.5-1.36)	0.84 (0.56-1.44)	0.82 (0.58-1.28)
		2-5 (n=14)	0.77 (0.41-1.48)	0.63 (0.36-0.85)	0.58 (0.36-0.9)	0.64 (0.39-1)	0.68 (0.37-1.02)
		5-7.5 (n=10)	0.68 (0.4-1.41)	0.58 (0.39-0.74)	0.41 (0.31-0.75)	0.46 (0.32-0.86)	0.55 (0.34-0.92)
		≥7.5 (n=12)	0.7 (0.43-1.54)	0.4 (0.31-0.59)	0.38 (0.2-0.59)	0.37 (0.2-0.69)	0.41 (0.24-0.61)
	**Season**						
		05-06	1.07 (0.56-2.67)	0.59 (0.39-0.8)	0.68 (0.4-0.93)	0.77 (0.41-1.12)	0.74 (0.38-1.08)
		06-07	0.88 (0.46-1.48)	0.54 (0.36-0.71)	0.51 (0.32-0.84)	0.62 (0.35-0.94)	0.58 (0.3-0.89)
		07-08	0.76 (0.5-1.57)	0.69 (0.4-0.83)	0.54 (0.38-0.78)	0.62 (0.41-0.94)	0.62 (0.38-0.81)
		08-09	0.71 (0.44-1.48)	0.57 (0.42-0.77)	0.62 (0.37-1.01)	0.66 (0.36-0.93)	0.68 (0.39-1.14)
		09-10	0.63 (0.43-1.17)	0.59 (0.36-0.85)	0.52 (0.31-1.25)	0.59 (0.31-1.38)	0.67 (0.37-1.14)
		10-11	0.61 (0.32-0.93)	0.5 (0.35-0.85)	0.38 (0.26-0.67)	0.38 (0.26-0.75)	0.43 (0.31-0.83)

^a^GFT: Google Flu Trends.

^b^COR: Pearson correlation coefficient.

^c^RMSE: root mean square error.

^d^MAPE: mean absolute percentage error.

**Table 4:** Mean rank and statistical significance from post hoc Nemenyi test. For each season-state combination, the model forms are ranked from best (rank=1) to worst (rank=8).

Measure	Model	Mean rank	GFT^a^	RRO	RRR	RRS	SS0	SRR	SRS
Pearson correlation coefficient (COR)	GFT	2.67							
	RR0	4.55	<.001						
	RRR	3.34	.002	<.001					
	RRS	3.68	<.001	<.001	.68				
	SS0	6.87	<.001	<.001	<.001	<.001			
	SRR	4.37	<.001	.98	<.001	.01	<.001		
	SRS	4.75	<.001	.97	<.001	<.001	<.001	.55	
	SSS	5.73	<.001	<.001	<.001	<.001	<.001	<.001	<.001
Root mean square error (RMSE)	GFT	4.46							
	RR0	5.27	.002						
	RRR	4.68	.96	.06					
	RRS	4.82	.62	.35	.99				
	SS0	5.77	<.001	.19	<.001	<.001			
	SRR	3.34	<.001	<.001	<.001	<.001	<.001		
	SRS	3.71	.005	<.001	<.001	<.001	<.001	.61	
	SSS	3.96	.2	<.001	<.001	<.001	<.001	.04	.92
Mean absolute proportion error (MAPE)	GFT	5.26							
	RR0	4.91	.65						
	RRR	5.18	.99	.89					
	RRS	5.7	.37	.002	.15				
	SS0	3.75	<.001	<.001	<.001	<.001			
	SRR	3.17	<.001	<.001	<.001	<.001	.07		
	SRS	3.93	<.001	<.001	<.001	<.001	.99	<.001	
	SSS	4.09	<.001	.001	<.001	<.001	.69	<.001	.99

^a^GFT: Google Flu Trends.

Results from Friedman-Nemenyi tests (see Table 4) show that SRS has the lowest mean rank for RMSE, and the difference is statistically significant from all other models, with the exception of SRR. SS0 has the lowest mean rank for MAPE but is not statistically different from either SRS or SRR. It is also interesting to note that models that continue to use ARIMA fit on regional ILI (SRR and SRS) match or outperform those that use ARIMA fit on state ILI (SS0 and SSS).

## Discussion

### Principal Findings

We described a method to nowcast ILI at subregional levels using GET and validated the developed models against real surveillance data across six influenza seasons and 50 states in the United States. The method was found to give improved estimates over an autoregressive model but underperformed relative to GFT. Variants of the method that used surveillance data at subregional levels, in a majority of the cases, bettered GFT.

Our results support earlier findings by other groups of the suitability of ARIMA models, both by themselves and in conjunction with other methods, in nowcasting ILI. This has particular relevance for very small settings, say a hospital or a rural county health department, where internal estimates of ILI are available and short-horizon forecasts are of interest for resource planning.

It was also found that data accessible through GET API are sparse at finer geographical granularity, and methods that rely solely on search trend data may not be viable for localized nowcasts. The inheritance method described here addresses the issue to some extent, as tests for the impact of inheritance on the models' performance found that inheritance improves correlation overall, particularly in states with low population; however, it has no significant impact on RMSE and increases MAPE (Multimedia Appendix 1; Figure S3). Additional analysis is necessary to identify scenarios, for example, when a state's signal is below a fraction of the parent region or below a threshold determined by historical likelihood, in which inheritance is useful. Incorporation of alternate data streams—such as electronic health records and social media—as additional features to the random forest models may obviate the need for inheritance and potentially improve nowcasts.

The reduced errors of the S* models, which use state-level ILI as the training response variable, make a case for the public release of this information every week. CDC estimates ILI at HHS regions by aggregating data submitted through the US Outpatient ILI Surveillance Network (ILINet) by about 2000 outpatient health care providers in the United States every week. Aggregation of data at subregional levels is possible in theory, but there are concerns about patient and provider privacy. However, given our findings that reliance on regional ILI with or without subregional GET produces inferior subregional nowcasts and that these are only marginally better than use of regional ILI as a proxy for subregional ILI, perhaps it is necessary to revisit specific concerns about privacy and to explore anonymization methods whose use might permit release of ILINet data at the subregional level.

As all states in an HHS region will have the same RRR nowcast estimate, the performance of RRR and GFT in nowcasting *regional* ILI can be compared. No significant difference was found between RRR nowcasts and GFT at the regional level for any of the three accuracy measures used (see Multimedia Appendix 1; Table S4). The superior performance of GFT over R* models at the state level, however, requires additional analysis. Although we have little information on the GFT model form, we believe that Google had no access to subregional CDC ILI data to train subregional models. As a consequence, GFT municipal- and state-level ILI estimates were likely extrapolations of regional models, akin to the R* models described here. This might also explain why our S* models outperform GFT in terms of RMSE and MAPE—by building models at the state level, biases in state level ILI data relative to the parent region were eliminated, thereby reducing error (this implicit bias correction is indeed observed; see Multimedia Appendix 1; Figure S4). If GFT had the same access to search trends as is now publicly available through GET, the superior GFT subregional nowcasts relative to R* models suggest that both the feature set and the learning method presented here need to be improved further. If, on the other hand, GFT had full (100%) access to GET, then its superior performance relative to R* models may stem more from that discrepancy in access.

One limitation of the validation method reported above is that it does not account for back-revisions to ILI data. CDC's ILI estimates are updated for multiple weeks following the week of initial release, as additional providers submit delayed data. We did not have access to information on how state-level ILI was updated over time but only to the final stable ILI. If this detailed versioned dataset were available, a more robust validation comparing nowcasts generated using transient estimates of ILI with the final stable ILI would have been possible.

### Conclusions

Overall, the findings suggest that nowcast extrapolation to more local scales are likely to remain challenging, as long as data at these scales remain restricted. As public health interventions and hospital planning can benefit from timely and localized estimates of ILI, relaxation of these restrictions may be warranted.

## Appendix

Multimedia Appendix 1 Supporting information.

## Abbreviations

API: application programming interface
ARIMA: autoregressive integrated moving average
CDC: Centers for Disease Control and Prevention
GET: Google Extended Trends
GFT: Google Flu Trends
HHS: US Department of Health and Human Services
ILI: influenza-like illness
ILINet: US Outpatient Influenza-like Illness Surveillance Network
IQR: interquartile range
MAPE: mean absolute percentage error
MMWR: Morbidity and Mortality Weekly Report
RMSE: root mean square error
