# Supplementary material for: Subregional Nowcasts of Seasonal Influenza Using Search Trends
Source: J Med Internet Res. 2017 Nov 6;19(11):e370. doi: 10.2196/jmir.7486 (PMC5696582; doi:10.2196/jmir.7486)
Supplement: Multimedia Appendix 1 [file jmir_v19i11e370_app1.pdf]

# Subregional Nowcasts of Seasonal Influenza Using Search Trends

Sasikiran Kandula[1*], Daniel J Hsu[2], Jeffrey Shaman[1]

[1]Department of Environmental Health Sciences, Columbia University, New York, NY

[2]Department of Computer Science, Columbia University, New York, NY

* sk3542@cumc.columbia.edu

# Supporting Information

## Model Formulation

Let $y^r_{1:w}$ denote the logit transformed ILI observations for region $r$ through week $w$; $x^{r,t}_{1:v}$ = [$qf(t, r, 1), qf(t, r, 2), \ldots, qf(t, r, v)$] the vector of logit transformed query fractions for term $t$ at HHS region $r$ through week $v$; and, Q the feature set of terms identified as explanatory variables. The predictor matrix with query fractions for all terms in Q is thus:

$$X^r_{1:v} = \begin{bmatrix} x^{r,1}_1 & \cdots & x^{r,|Q|}_1 \\ \vdots & \ddots & \vdots \\ x^{r,1}_v & \cdots & x^{r,|Q|}_v \end{bmatrix}.$$

Due to the lag in ILI release, $v \geq w + 1$. We fit an ARIMA model using observations through weeks $w$ and forecast ahead through week $v$:

$$\tilde{y}^r_{1:v} = ARIMA(y^r_{1:w}).$$

The ARIMA result is added as an additional explanatory variable to the predictor matrix, yielding

$$\tilde{X}^r_{1:v} = \begin{bmatrix} X^r_{1:v} & {\tilde{y}^r_{1:v}}^T \end{bmatrix}.$$

Using $\tilde{X}^r_{1:w}$ as the predictor matrix and ${y^r_{1:w}}^T$ as the vector of responses, we train a random forest model, $\hat{f}^r_w$, for region $r$ at week $w$:

$${\hat{y}^r_{1:w}}^T = \hat{f}^r_w\left(\tilde{X}^r_{1:w}\right).$$

For a state $s$ in region $r$ with a query fraction matrix $X^s_{1:v}$, we append region $r$'s ARIMA results and use this as a test set with $\hat{f}^r_w$. Therefore the nowcast estimates of ILI for state $s$ are

$$\hat{y}^s_{(w+1):v} = \hat{f}^r_w\left(\tilde{X}^s_{(w+1):v}\right) \text{ where}$$

$$\tilde{X}^s_{1:v} = \begin{bmatrix} X^s_{1:v} & {\tilde{y}^r_{1:v}}^T \end{bmatrix}$$

We refer to this form as RRS. Hence, the alternate model form RR0, where the state's nowcast is simply its region's ARIMA estimate is: $\hat{y}^s_{(w+1):v} = \tilde{y}^r_{(w+1):v}$, and the model form RRR, where the state's GET query fractions are replaced with the query fractions of its parent region is: $\hat{y}^s_{(w+1):v} = \hat{f}^r_w\left(\tilde{X}^r_{(w+1):v}\right)$

## Alternate model forms – state ILI as response

Let $y^s_{1:w}$ and $X^s_{1:v}$ be defined analogous to $y^r_{1:w}$ and $X^r_{1:v}$ respectively. We fit an ARIMA model using state-level ILI. $\tilde{y}^s_{1:v} = ARIMA(y^s_{1:w})$

**SS0**: $\hat{y}^s_{(w+1):v} = \tilde{y}^s_{(w+1):v}$

**SSS:** $\widetilde{Xa}^s_{1:v} = \left[X^s_{1:v} \quad {\tilde{y}^s_{1:v}}^T\right]$

$${\hat{y}^s_{1:w}}^T = \widehat{fa}^s_w\,(\widetilde{Xa}^s_{1:w})$$

$$\hat{y}^s_{(w+1):v} = \widehat{fa}^s_w\,(\widetilde{Xa}^s_{(w+1):v})$$

**SRS:** $\widetilde{Xb}^s_{1:v} = \left[X^s_{1:v} \quad {\tilde{y}^r_{1:v}}^T\right]$

$${\hat{y}^s_{1:w}}^T = \widehat{fb}^s_w\,(\widetilde{Xb}^s_{1:w})$$

$$\hat{y}^s_{(w+1):v} = \widehat{fb}^s_w\,(\widetilde{Xb}^s_{(w+1):v})$$

**SRR:** $\widetilde{Xc}^s_{1:v} = \left[X^r_{1:v} \quad {\tilde{y}^r_{1:v}}^T\right]$

$${\hat{y}^s_{1:w}}^T = \widehat{fc}^s_w\,(\widetilde{Xc}^s_{1:w})$$

$$\hat{y}^s_{(w+1):v} = \widehat{fc}^s_w\,(\widetilde{Xc}^s_{(w+1):v})$$

**Figure S1.** GET query fractions for select terms at US national level, high- (CA) and low population (WA) states and large (New York, NY) and medium sized cities (Seattle, WA)

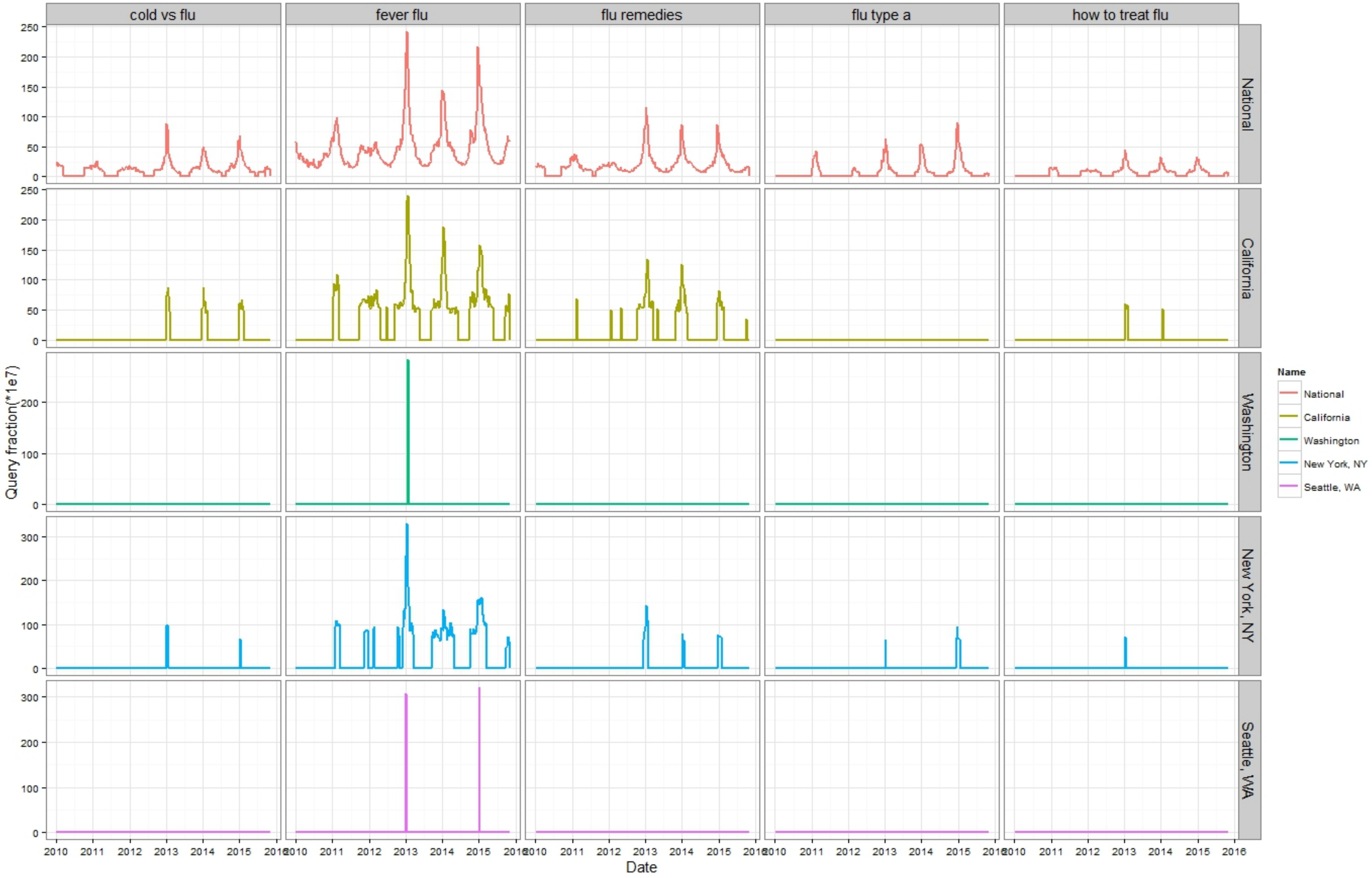

## Sensitivity Analysis of the choice of floor

For the analysis reported in the manuscript, query fractions that were zeros were replaced by a very small value (1E-12) before the logit transformation was applied. We performed a sensitivity analysis on the choice of floor by testing two alternative floor values - 1E-8 and 1E-10. Figure S2 shows that COR/RMSE/MAPE are virtually unchanged for all model variants during the 6 seasons for any of the three values of floor. Additionally, we performed a Friedman-Nemenyi test and established that these differences are not statistically significant.

**Figure S2.** Value of measures (COR, MAPE, RMSE) for each season and model variant using the three alternative floors: Base (1E-12) as reported in the manuscript, Floor10 (1E-10) and Floor8( 1E-8)

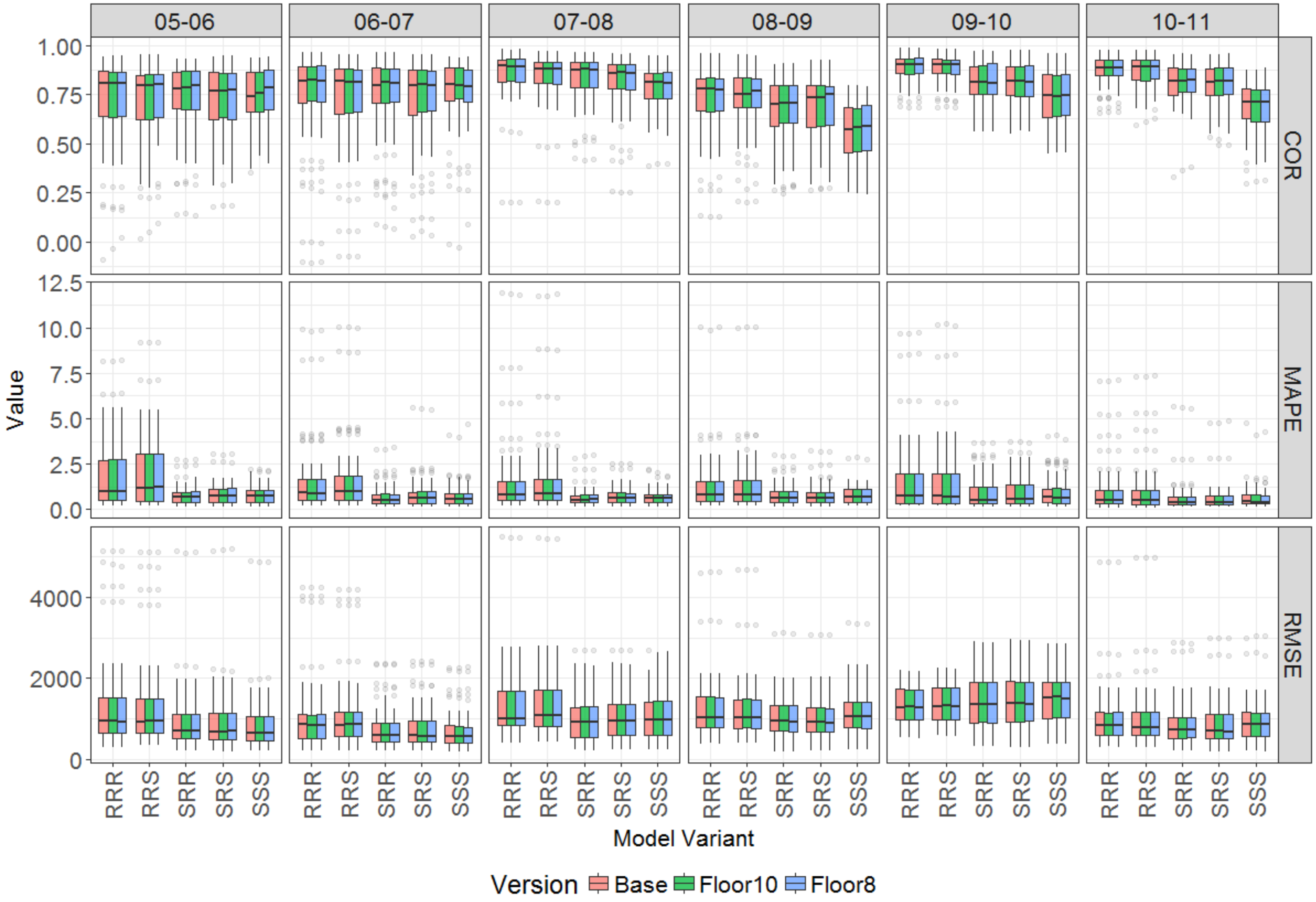

## Analysis of the effect of inheritance on nowcast quality

For the three model variants that inherit regional query fractions (RRS, SRS and SSS), we re-estimated nowcasts without using inheritance i.e. the state query fractions were unaltered. We compared the quality of these estimates with estimates that result from the use of inheritance. In Figure S3, we see that inheritance improves correlation overall and particularly in low population states, but has no significant impact on root mean squared error and increases mean absolute proportion error. We performed paired Wilcoxon tests to test for significance and found that the differences in correlation and MAPE are significant but not with RMSE.

**Figure S3.** Comparison of nowcasts using inheritance (Base) and nowcasts without inheritance (No.Inherit) (a) Correlation; (b) Correlation in states with low population; (c) Root mean Squared Error; and (d) Mean Absolute Proportional Error

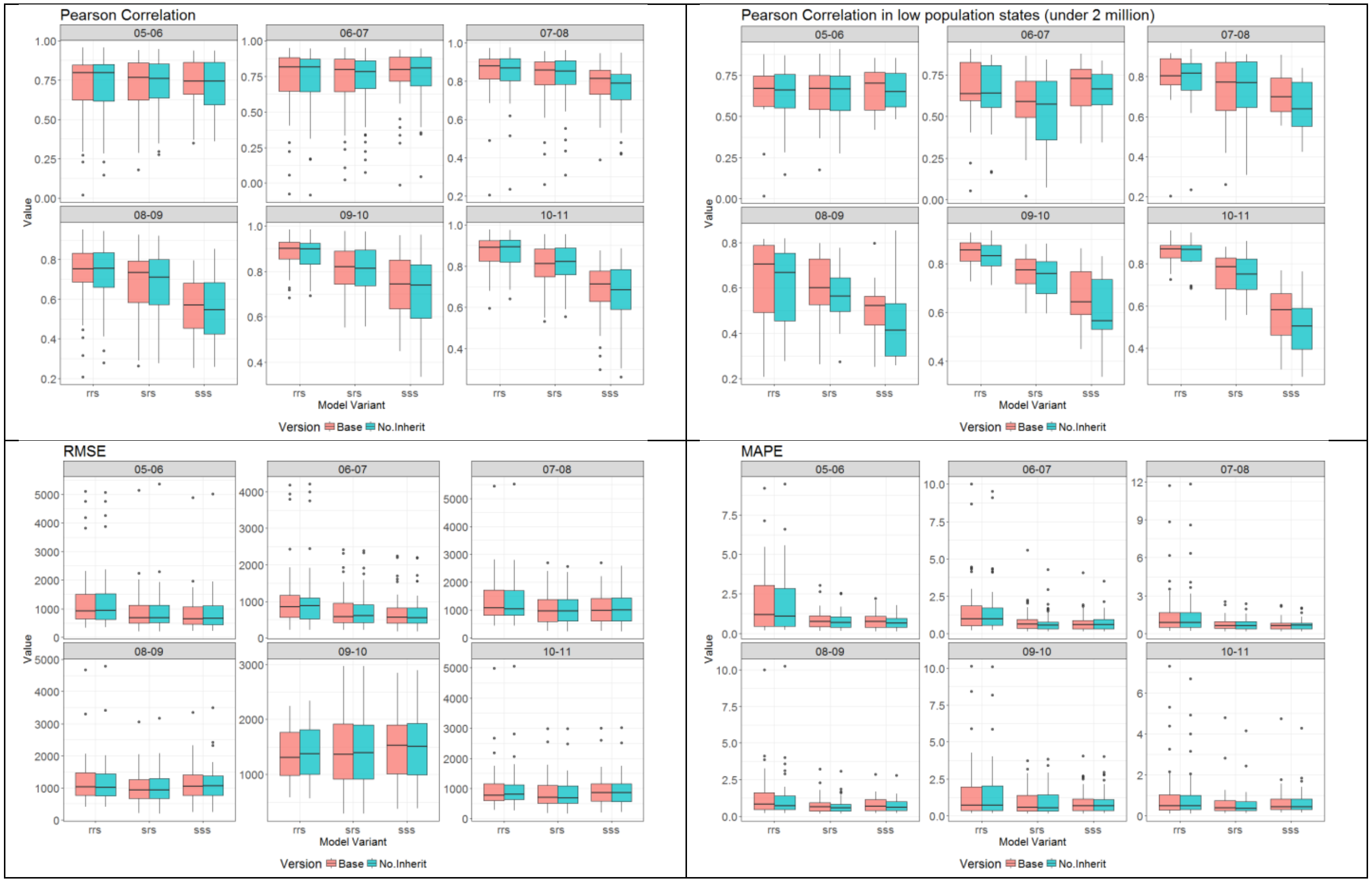

**Figure S4.** Figure shows the nowcast estimate for Arizona for 05-06 through 10-11 influenza season. The true ILI are in black and Google Flu Trends estimate is in red. The models built using regional ILI(R* models) consistently overestimate ILI which is corrected in models built with state's ILI (S* models)

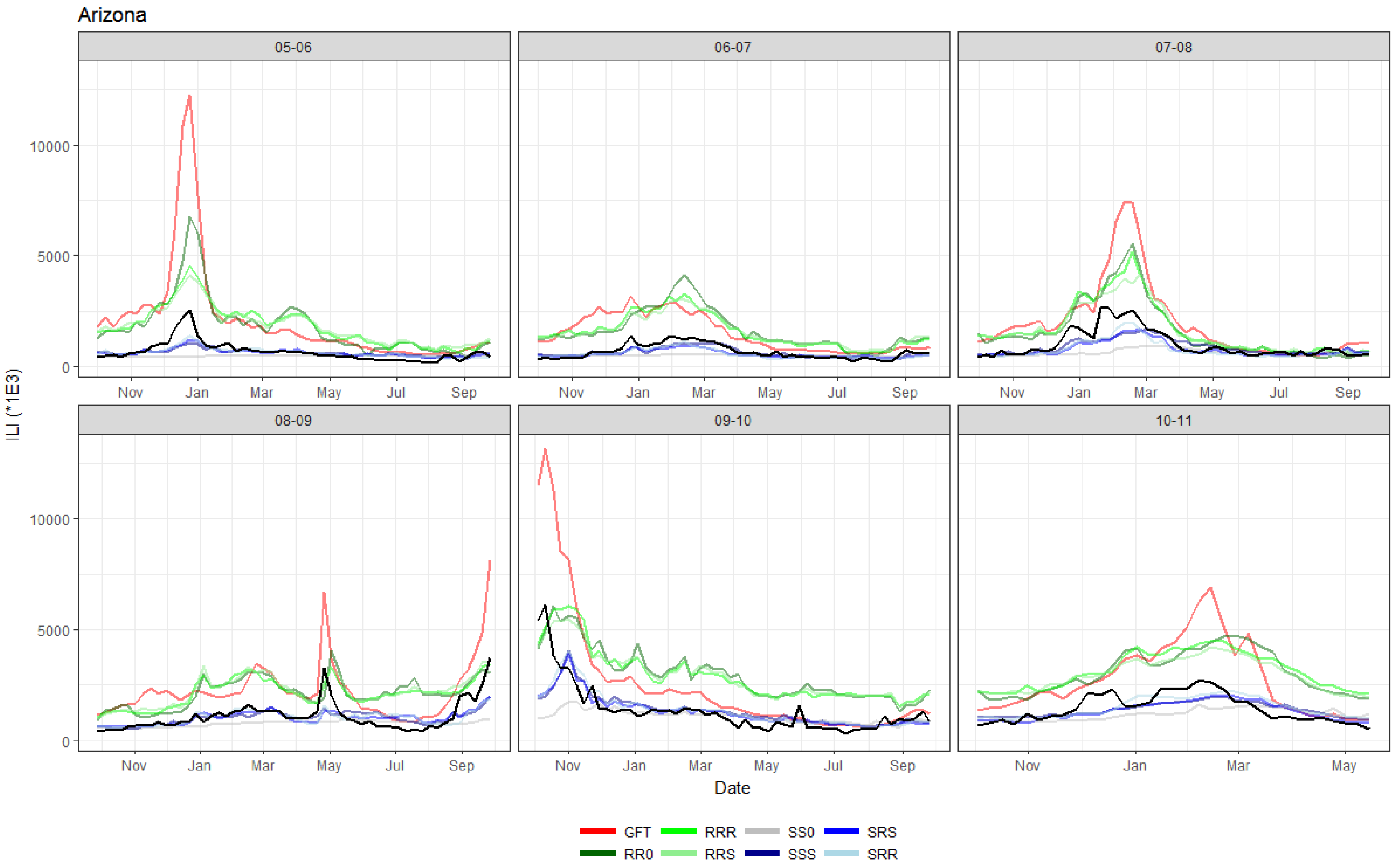

**Figure S5** Pearson Correlation coefficient disaggregated by state's population size and season

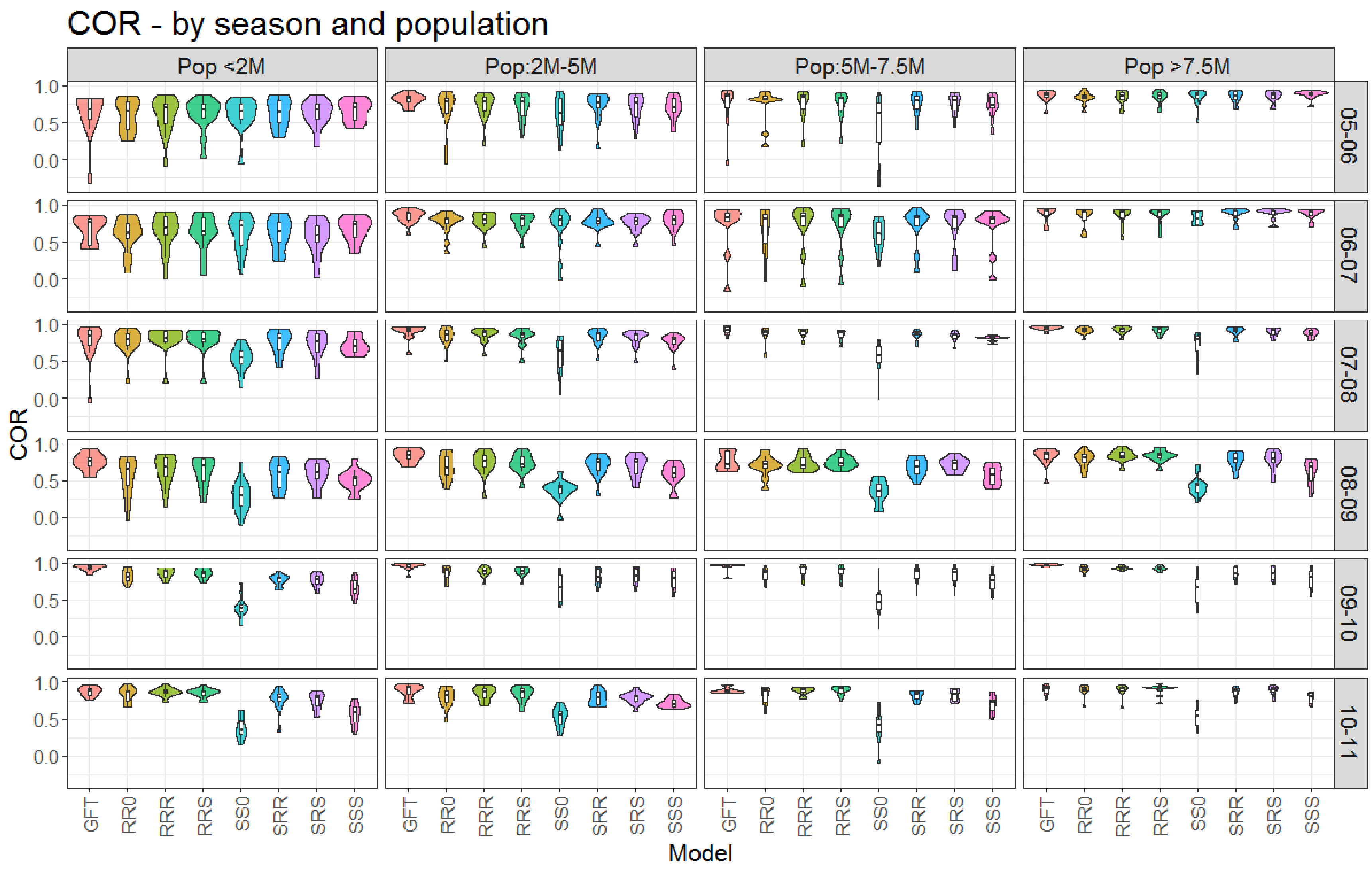

**Figure S6** RMSE disaggregated by state's population size and season

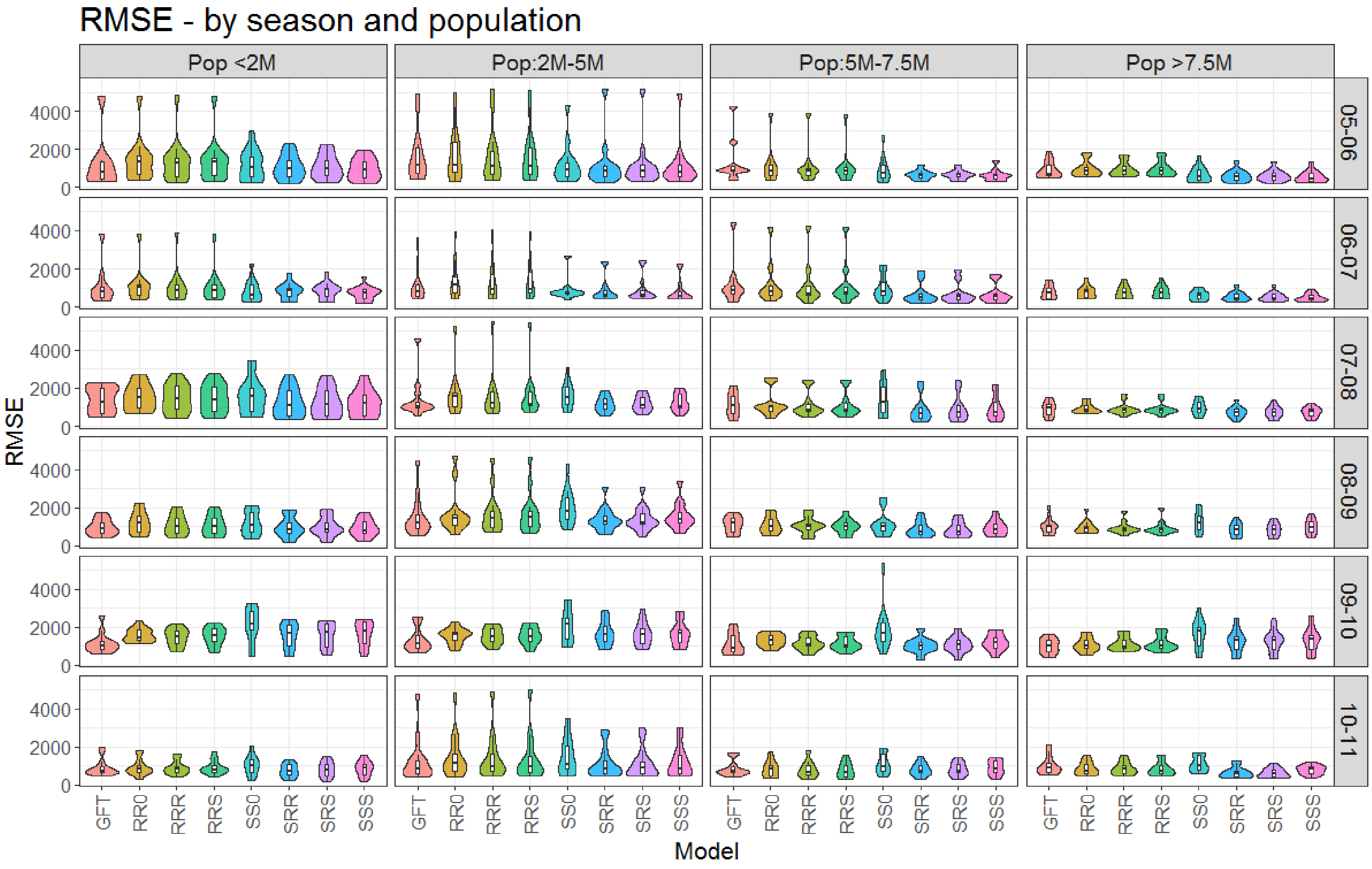

**Figure S7** Log transformed MAPE disaggregated by state's population size and season

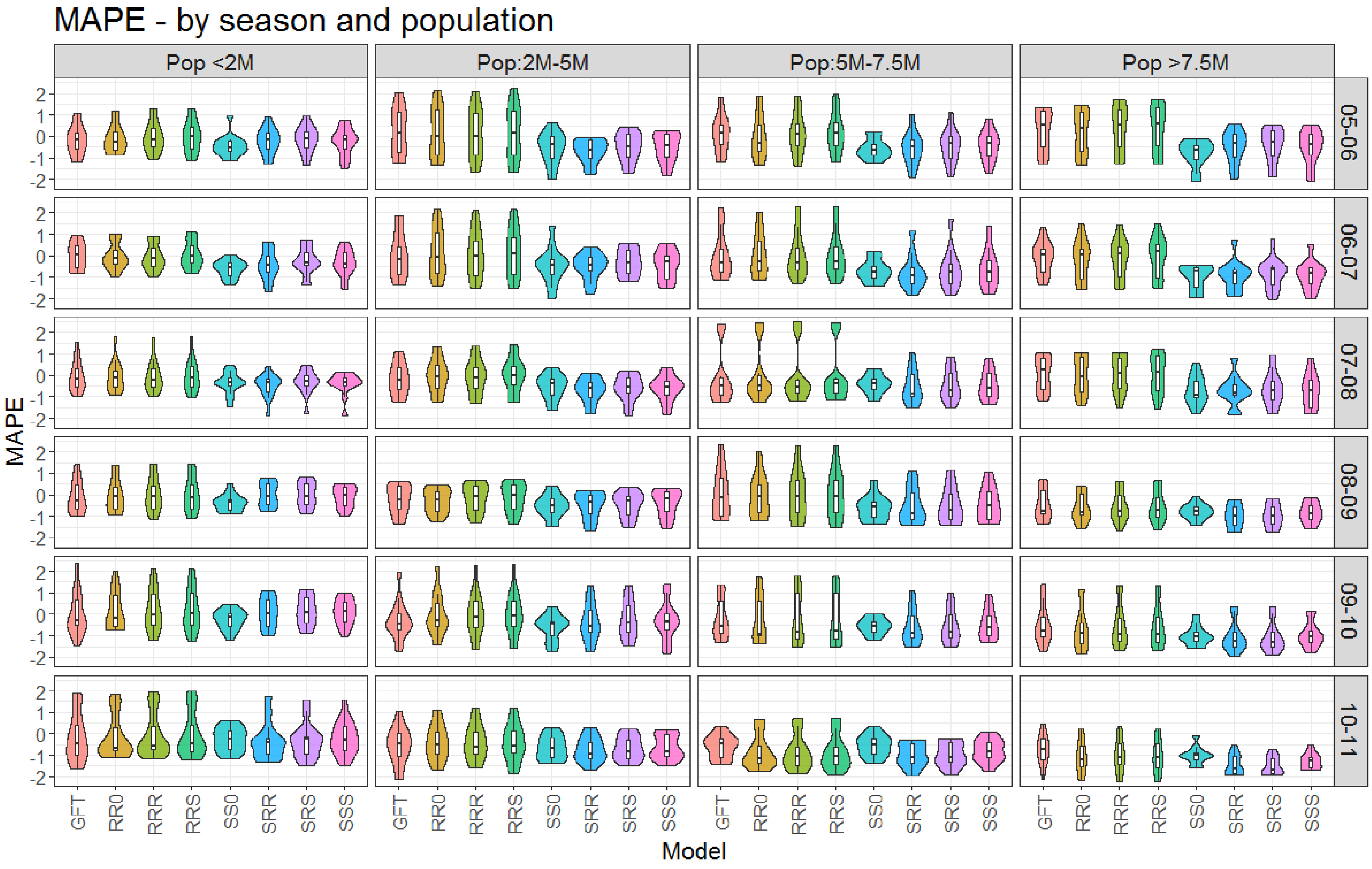

**Table S1. For each model variant, the response variance and query fractions used to build the models.**

| | Response | ARIMA trained on | Query fractions |
|---|---|---|---|
| **RR0** | ILI - Regional | ILI - Regional | |
| **RRR** | ILI - Regional | ILI - Regional | GET - Regional |
| **RRS** | ILI - Regional | ILI - Regional | GET - State |
| **SSO** | ILI - State | ILI - State | |
| **SRR** | ILI - State | ILI - Regional | GET - Regional |
| **SRS** | ILI - State | ILI - Regional | GET - State |
| **SSS** | ILI - State | ILI - State | GET - State |

We performed paired Wilcoxon signed-rank test (1, 2) to check if there is a statistically significant difference in the mean of the measures (COR, RMSE and MAPE) between pairs of model forms, to complement Friedman/Nemenyi tests reported in the manuscript.

**Table S2. P-values for Wilcoxon ranked sum test for pairs of model forms. A two-tailed test is performed followed by tests for two alternative hypotheses: a) above diagonal: row < column; and b) below diagonal: column > row.**

| | COR | | | | RMSE | | | | MAPE | | | |
|---|---|---|---|---|---|---|---|---|---|---|---|---|
| | **RR0** | **RRR** | **RRS** | **GFT** | **RR0** | **RRR** | **RRS** | **GFT** | **RR0** | **RRR** | **RRS** | **GFT** |
| **RR0** | | <.001 | <.001 | <.001 | | .93 | .96 | 1 | | <.001 | <.001 | .34 |
| **RRR** | 1 | | .73 | <.001 | .07 | | .63 | 1 | 1 | | .98 | 1 |
| **RRS** | 1 | .27 | | <.001 | .04 | .37 | | 1 | 1 | .02 | | 1 |
| **GFT** | 1 | 1 | 1 | | <.001 | <.001 | <.001 | | .66 | <.001 | <.001 | |

**Table S3. P values for Wilcoxon ranked sum test for pairs of model forms. NS indicates no significant difference in means. A two-tailed test is performed followed by tests for two alternative hypotheses: a) above diagonal: row < column; and b) below diagonal: column > row.**

| | COR | | | | | RMSE | | | | | MAPE | | | | |
|---|---|---|---|---|---|---|---|---|---|---|---|---|---|---|---|
| | **SS0** | **SRR** | **SRS** | **SSS** | **GFT** | **SS0** | **SRR** | **SRS** | **SSS** | **GFT** | **SS0** | **SRR** | **SRS** | **SSS** | **GFT** |
| **SSO** | | <.001 | <.001 | <.001 | <.001 | | 1 | 1 | 1 | 1 | | <.001 | <.001 | <.001 | <.001 |
| **SRR** | 1 | | .09 | 1 | <.001 | <.001 | | 1 | <.001 | <.001 | 1 | | 1 | .12 | <.001 |
| **SRS** | 1 | .91 | | 1 | <.001 | <.001 | .001 | | <.001 | <.001 | 1 | .001 | | .001 | <.001 |
| **SSS** | 1 | <.001 | <.001 | | <.001 | <.001 | 1 | 1 | | .01 | 1 | .88 | 1 | | <.001 |
| **GFT** | 1 | 1 | 1 | 1 | | <.001 | 1 | 1 | .99 | | 1 | 1 | 1 | 1 | |

**Table S4. Mean rank and statistical significance from posthoc Nemenyi test. For each region-season combination the model forms are ranked from best (rank = 1) to worst (rank = 3). For each pair of model forms, the actual P values are reported except when P < .001 (indicated by ***).**

| | COR | | | RMSE | | | MAPE | | |
|---|---|---|---|---|---|---|---|---|---|
| | **Mean Rank** | **GFT** | **RRO** | **Mean Rank** | **GFT** | **RRO** | **Mean Rank** | **GFT** | **RRO** |
| **GFT** | 1.58 | | | 2.12 | | | 2.25 | | |
| **RR0** | 2.45 | *** | | 2.07 | .97 | | 1.52 | *** | |
| **RRR** | 1.97 | .09 | .02 | 1.8 | .21 | .31 | 2.23 | .99 | *** |

**Table S5. List of entities and terms used to query GET for search frequencies**

| | |
|---|---|
| **Entities** | 1918 influenza (/m/01c751), 2009 influenza (/m/05zs0_7), 2009 influenza vaccine (/m/065yz34), amantadine (/m/048pyy), antiviral drug (/m/0d3p4), aspirin (/m/0qkc), avian influenza (/m/0292d3), body aches (/m/013677), body temperature (/m/026_p_h), bronchitis (/m/047gmsk), chills (/m/02mdc7), cold (/m/0n073), cough (/m/01b_21), cough medicine (/m/01nf88), fever (/m/0cjf0), flu shot (/m/0416v7), guaifenesin (/m/03pnl6), h1n1 (/m/087t7g), h5N1 (/m/03zx0w), headache (/m/0j5fv), ibuprofen (/m/014d3g), infection (/m/098s1), influenza-like illness (/m/05_5py4), influenza (/m/0cycc), influenza virus a (/m/028tns), influenza virus b (/m/0b2cnj), malaise (/m/0418s3), oseltamivir (/m/03t8j0), otalgia (/m/05vywy), pandemic (/m/0899nb), pharyngitis (/m/01gkcc), pneumonia (/m/0dq9p), rapid influenza test (/m/09gh4jl), rhinitis (/m/02mdz9), rhinovirus (/m/0q4zv), robitussin DAC (/m/0412lbl), RSV (/m/02f84_), runny nose (/m/06p_bp), SARS (/m/01byzl), sick day (/m/0h47fv), sinusitis (/m/072hv), strep throat (/m/0mztl), swine flu (/m/057c6k), tylenol (/m/0lbtm) |
| **Terms** | afrin, baby cough, benzonatate, body temperature, bronchitis, child fever, cold and flu, cold or flu, cold remedies, cold symptoms, cold vs flu, common cold, cough and cold, cough fever, cough medicine, coughing, coughing up, cure flu, cure for flu, cure the flu, delsym, dry cough, feed a cold, fever flu, fever in children, fever temperature, flu and cold, flu care, flu children, flu fever, flu how long, flu how long contagious, flu in adults, flu incubation, flu kids, flu or cold, flu pneumonia, flu remedies, flu report, flu sore throat, flu stomach, flu symptoms children, flu symptoms fever, flu symptoms in children, flu temperature, flu type a, flu virus, flu vs cold, how long does the flu last, how long flu, human temperature, incubation period, influenza, influenza a, influenza b, influenza symptoms, is bronchitis, low body temperature, low temperature, nasal congestion, nyquil, oscillococcinum, pneumonia contagious, pneumonia symptoms, pneumonia treatment, robitussin, robitussin dm, sinus infection, sinusitis, starve a cold, stomach flu, stop coughing, strep throat, strep throat symptoms, symptoms of flu, symptoms of sinus infection, tamiflu side effects, temperature fever, the flu, the flu virus, the flue, toddler cough, treat flu, tussionex, tylenol cold, type a flu, upper respiratory, upper respiratory infection, viral flu, walking pneumonia |

## References


1. Wilcoxon F. Individual comparisons by ranking methods. Biometrics bulletin. 1945;1(6):80-3.
2. Bauer DF. Constructing confidence sets using rank statistics. Journal of the American Statistical Association. 1972;67(339):687-90.
